# Reliability of CEUS and MRI for grading knee-joint inflammation in juvenile idiopathic arthritis

**DOI:** 10.1007/s00330-026-12627-z

**Published:** 2026-05-15

**Authors:** Sílvia Costa Dias, Catarina Costa Ferreira, Inês Santos, Miguel Castro, Mariana Rodrigues, Francisca Aguiar, Cláudia Camila Dias, Bac Nguyen, Vitor Silva, Isabel Ramos, Damjana Ključevšek, Iva Brito, Karen Rosendahl

**Affiliations:** 1 Department of Medicine, Faculty of Medicine of the University of Porto (FMUP), Porto, Portugal; 2 Department of Radiology, University Hospital Centre of São João Porto (CHUSJ), Porto, Portugal; 3 Rheumatology Unit, Unidade Local Saúde Viseu Dão-Lafões, Viseu, Portugal; 4 Paediatric and Young Adult Rheumatology Unit, University Hospital Centre of São João (CHUSJ), Porto, Portugal; 5 Knowledge Management Unit, Faculty of Medicine of the University of Porto (FMUP), Porto, Portugal; 6 CINTESIS@RISE, Department of Community Medicine, Information and Health Decision Sciences (MEDCIDS), Faculty of Medicine of the University of Porto (FMUP), Porto, Portugal; 7 Division of Radiology and Nuclear Medicine, Oslo University Hospital, Rikshospitalet, Oslo, Norway; 8 Department of Radiology, University Children’s Hospital Ljubljana, Ljubljana, Slovenia; 9 Department of Clinical Medicine, Faculty of Health Sciences, UiT the Arctic University of Norway, Tromsø, Norway; 10 Section of Paediatric Radiology, University Hospital of North Norway, Tromsø, Norway

**Keywords:** Synovitis, Arthritis, Knee, Ultrasonography, Magnetic resonance imaging

## Abstract

**Objective:**

To examine the reliability of contrast-enhanced MRI (CEMRI) and contrast-enhanced ultrasound (CEUS) for grading arthritis.

**Materials and methods:**

Prospective study on 1–18-year-olds with juvenile idiopathic arthritis (JIA), performed during 2023/2024. All had CEUS and CEMRI of the knee on the same day. Two observers independently scored the US-video-clip for inflammation (0–3 scale), with a second reading after 6 weeks. Using VueBox®, peak enhancement (PE), time to peak (TTP) and wash-in area under the curve (WiAUC) were measured by both observers, separately. The MRIs were scored for inflammation (0–3 scale) twice by two observers. Peak enhancement intensity (PEI), TTP and initial area under the curve (iAUC) (Syngo.via®) were measured.

**Results:**

Fifty-two patients (35 females), median age 12.4 years (IQR 8.9–15.6 years), were included. CEUS showed almost perfect intra/interreader agreement for the degree of overall inflammation (intrareader kappa 0.90 [0.83–0.98], interreader kappa 0.85 [0.76–0.93]). Interreader PE and WiAUC agreement was excellent (ICC 0.99 [95% CI 0.99–1.00] and 0.97 [95% CI 0.95–0.98], respectively), while TTP performed moderately (ICC 0.66 [95% CI 0.40–0.80]). On CEMRI, intra/interreader agreement for overall synovial inflammation was almost perfect (intrareader kappa 0.94 [95% CI 0.88–1.00]; interreader kappa 0.92 [95% CI 0.85–1.00]). Interreader PEI and iAUC agreement was excellent (ICC 0.99 [95% CI 0.98–0.99] and 0.98 [95% CI 0.97–0.99], respectively), while TTP had good agreement (ICC 0.76 [95% CI 0.59–0.86]).

**Conclusion:**

Subjective grading of synovial inflammation on a 0–3 scale demonstrated high reliability for both CEUS and CEMRI. Both methods have excellent relative reliability (ICC) for quantitative measurements; however, their absolute precision (LoA) is poor.

**Key Points:**

***Question*** *Are CEUS and CEMRI reliable tools to grade the activity of knee-joint arthritis in children with juvenile idiopathic arthritis?*

***Findings*** *Subjective grading of arthritis performed well within and between observers for both CEUS and CEMRI. Both methods provide reliable dynamic measurements; however, their precision is poor.*

***Clinical relevance*** *CEUS offers high-precision, real-time imaging for grading active knee-joint inflammation in children with juvenile idiopathic arthritis, with future potential to replace the current standard of CEMRI.*

**Graphical Abstract:**

**Figure:**
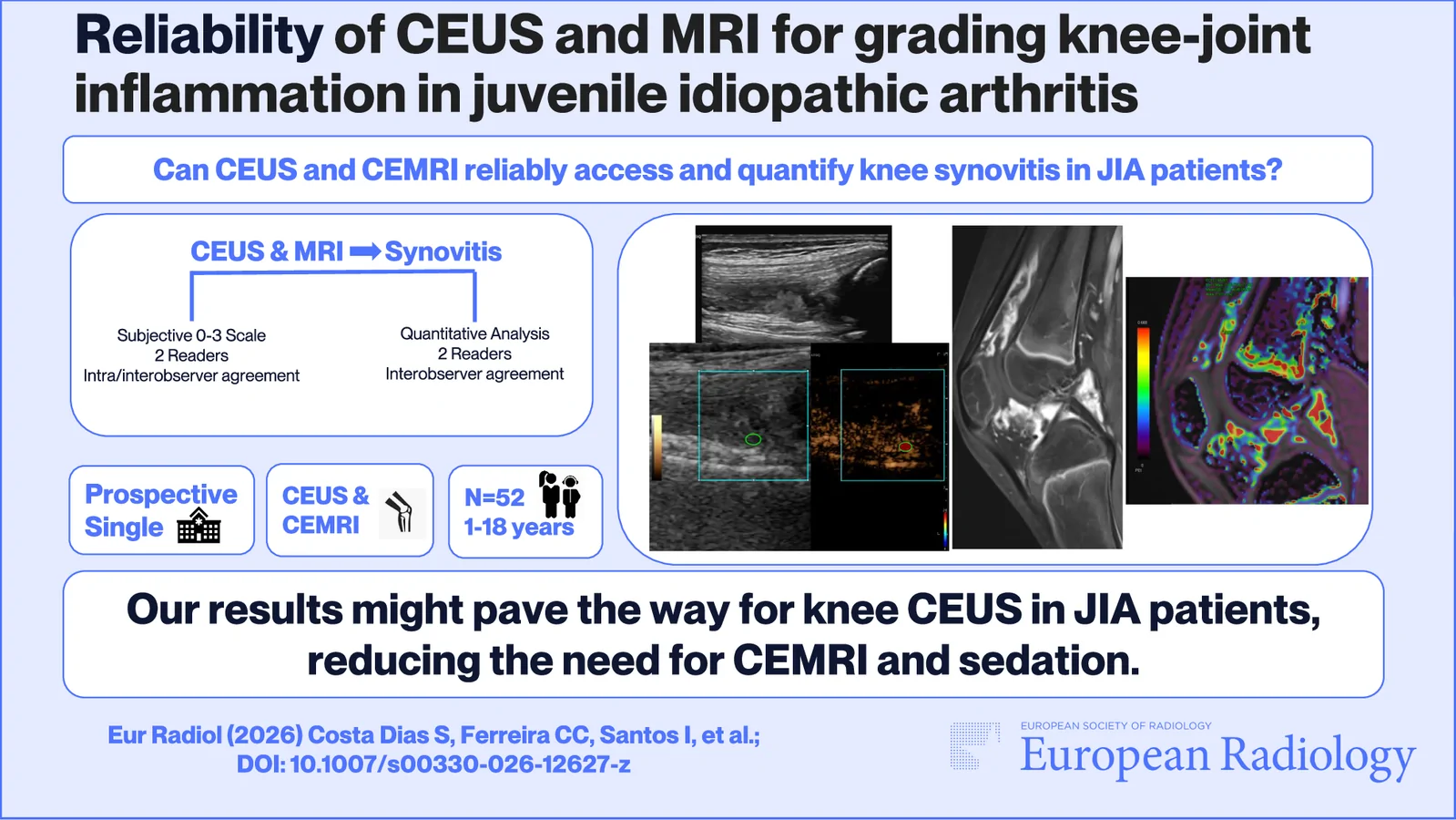

## Introduction

Juvenile idiopathic arthritis (JIA) is a heterogeneous disease including all forms of chronic arthritis of unknown origin and with onset before 16 years of age [1, 2]. It affects 1–2 in 1000 children (f:m 3:1) and is characterised by chronic synovial inflammation, with risk of developing progressive joint destruction [3]. Although modern treatment has reduced the morbidity, children are still faced with periods of active disease, pain and reduced mobility, often continuing into adulthood [4, 5].

The diagnosis is based on history, clinical and laboratory findings, with imaging emerging as an important tool narrowing differential diagnosis, monitoring disease activity and detecting treatment complications [6]. While radiography still plays a role in joint damage evaluation and secondary growth disturbances, US and MRI are the preferred methods for assessment of disease activity [6]. Several examination techniques, protocols and grading systems for inflammatory change of the knee joint have been suggested for both US and MRI; however, the literature on their reliability is limited, including two papers on MRI with 25 patients each [7, 8] and none on US.

This study aimed to evaluate the reliability of contrast-enhanced ultrasound (CEUS) and contrast-enhanced MRI (CEMRI) for assessing knee synovitis in JIA, both subjectively (0–3 scale) and quantitatively (for CEUS: peak enhancement [PE], time to peak [TTP], wash-in area under the curve [WiAUC]; for CEMRI: peak enhancement intensity [PEI], TTP, initial area under the curve [iAUC]).

Finally, we aimed to assess the reliability of the inflammatory components of the juvenile arthritis MRI scoring (JAMRIS) system for the knee, a system also including assessment of bone marrow oedema [7].

## Materials and methods

This is part of a prospective, single-centre study on children with a known diagnosis of JIA, performed during 2023–2024. Participants were consecutively recruited from the Paediatric Rheumatology Clinic at the University Hospital Centre of São João Porto (CHUSJ). Children aged 1–18 years, meeting the ILAR criteria [9] for JIA, with current or previous knee involvement, were included. Excluded were children with a history of intra-articular steroid injection or trauma to the knee within the last 2 months.

For this reliability study, we used demographic and CEUS/CEMRI data. The study was approved by the Regional Ethics Committee (number 282-20), and written informed consent was obtained from each participant and/or their caregiver, according to national guidelines.

### Imaging techniques

All children had a CEUS examination of the affected knee, with an additional CEMRI performed later on the same day, at an interval of at least 60 min, both with the child supine and 30° knee flexion. Intravenous access using a 20 G catheter was secured via an antecubital vein and used for both examinations. According to the European Medicines Agency [10], almost 100% of the microbubbles in SonoVue are eliminated through the lungs within 15 min. No general anaesthesia was given.

The ultrasound examinations were performed by one examiner with 12 years of experience in paediatric radiology, using a Logiq E10 machine (Version R3, General Electric®) with a linear 6–15 MHz transducer and defined pre-sets, according to a standardised protocol [11]. Sagittal scans through the suprapatellar recess were performed to identify the most prominent and hyperaemic synovium (Colour/Power/Micro Doppler). For the CEUS, a vascular linear 2–10 MHz transducer, with an adjusted musculoskeletal contrast preset, was used. A 3-way intravenous cannula was adapted to the catheter. Next, a bolus of Sulfur Hexafluoride (Sonovue®) contrast was injected; 2.4 mL to patients < 50 kg and 4.8 mL to those > 50 kg, followed by 10 mL saline chaser. Thereafter, a 2-min continuous, sagittal acquisition was performed in the same position.

The MRI examinations were performed on a 3-Tesla scanner (Siemens® Magnetom Vida) by a specially trained MRI radiographer, using an 18-channel phased-array knee transmit/receive (Tx/Rx) coil, according to a predefined protocol (Supplemental Material). A cushion (Multipad, Pearl Technology) was used to immobilise the knee. Gadolinium (Gadobutrol—Gadovist®, Bayer) was injected with a pump (0.1 mmol/Kg) at a rate of 1–3 mL/s, according to the size of the needle.

For this reliability study, we used the T1 vibe Dixon fat-saturated (FS) pre-gadolinium and T1 vibe Dixon FS controlled aliasing in parallel imaging results in higher acceleration (CAIPIRINHA) first 2 min with gadolinium (isotropic) and T1-w TSE FS post-gadolinium sagittal and axial series.

### Image analysis

#### CEUS

##### Subjective assessment of synovial inflammation on a 0–3 scale.

**Fig. 1:**
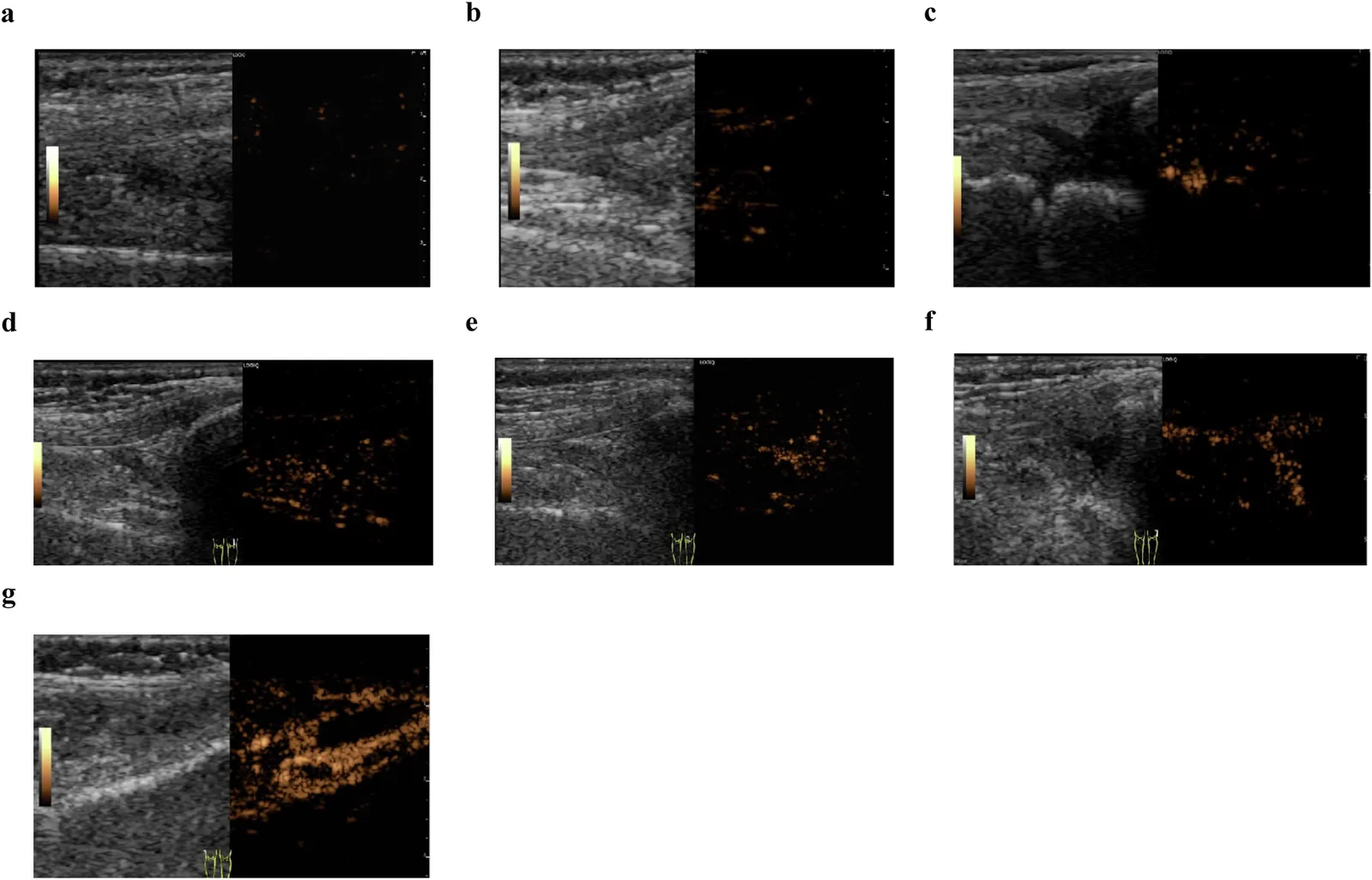
CEUS atlas, with examples of the suprapatellar recess of the knee, longitudinal view. Sofware of VueBox® with Dual-screen mode with simultaneous display of grey-scale image (left) and contrast-only image (right) in the same view: **a**, **b** Grade 0 (few bubbles allowed); **c**, **d** Grade 1 (mild); **e**, **f** Grade 2 (moderate); **g** Grade 3 (severe)

All CEUS videoclips (0–120 s) were anonymised, archived and reviewed retrospectively. Two observers independently and blinded to additional information, scored the degree of contrast enhancement on a 0–3 scale, adjusted from Klauser et al [12, 13]: grade 0 (few bubbles in the synovium); grade 1 (mild; patchy, synovial enhancement, intensity lower than surrounding tissue); grade 2 (moderate; more homogenous and diffuse synovial enhancement, intensity equal/slightly > surrounding tissue) or grade 3 (severe; fast/intense, homogenous synovial enhancement, intensity > surrounding tissue). An atlas (one still image from the 2-min videoclips—not later used for quantitative analysis—of seven patients) was devised to illustrate the 0–3 scale used (Fig. 1). A repeat grading (videoclips differently ordered) was performed after 6 weeks by both observers, independently.

##### Dynamic CEUS—quantitative assessment of synovial inflammation.

**Fig. 2:**
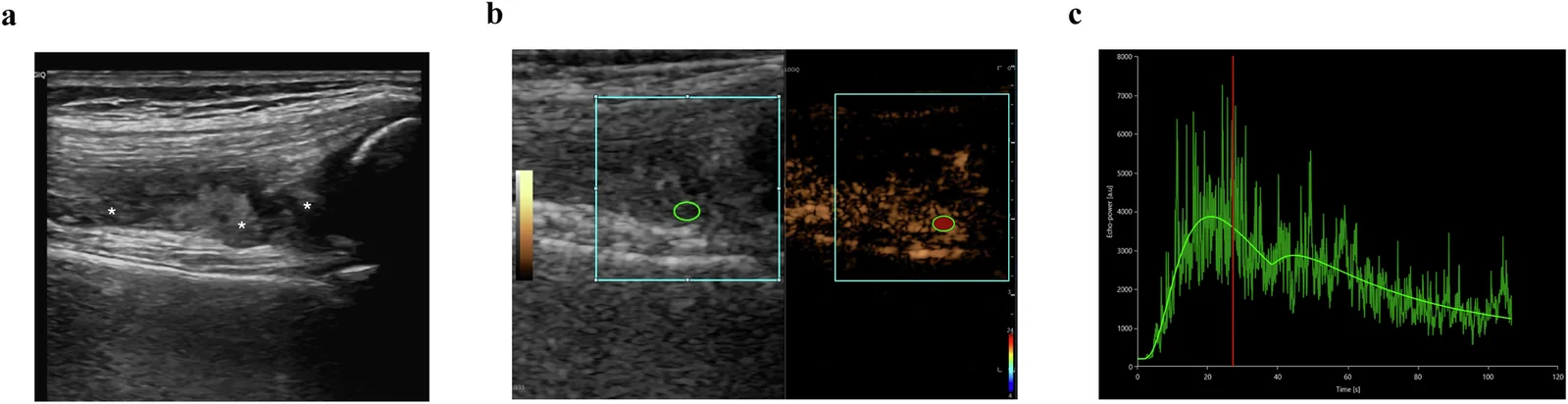
CEUS of the knee in a 7-year-old girl with JIA: **a** B-mode, sagittal view through the suprapatellar recess, showing severe synovial thickening (*); **b** CEUS, dual-screen mode with simultaneous display of grey-scale image (left) and contrast-only image (right) in the same view, showing the enhancing synovium with a region of interest (ROI) placed within (software VueBox); **c** corresponding time-intensity curve of the synovial enhancement during the first 120 s within the ROI

The DICOM video clip was post-processed with the CEUS quantification software VueBox® (version 7.5; Bracco Imaging). A sagittal frame showing the most prominent synovial membrane was selected in consensus by the two observers. Based on an applied colour intensity map, each observer chose three different regions of interest (ROIs), with a predefined saved standardised ROI (approximately 3.2–4.2 mm^2^, depending on the original size of the US image) for measurement of the most enhancing synovium (Fig. 2). PE, TTP and WiAUC were measured by both observers, separately.

#### MRI

##### Subjective assessment of synovial inflammation.

**Fig. 3:**
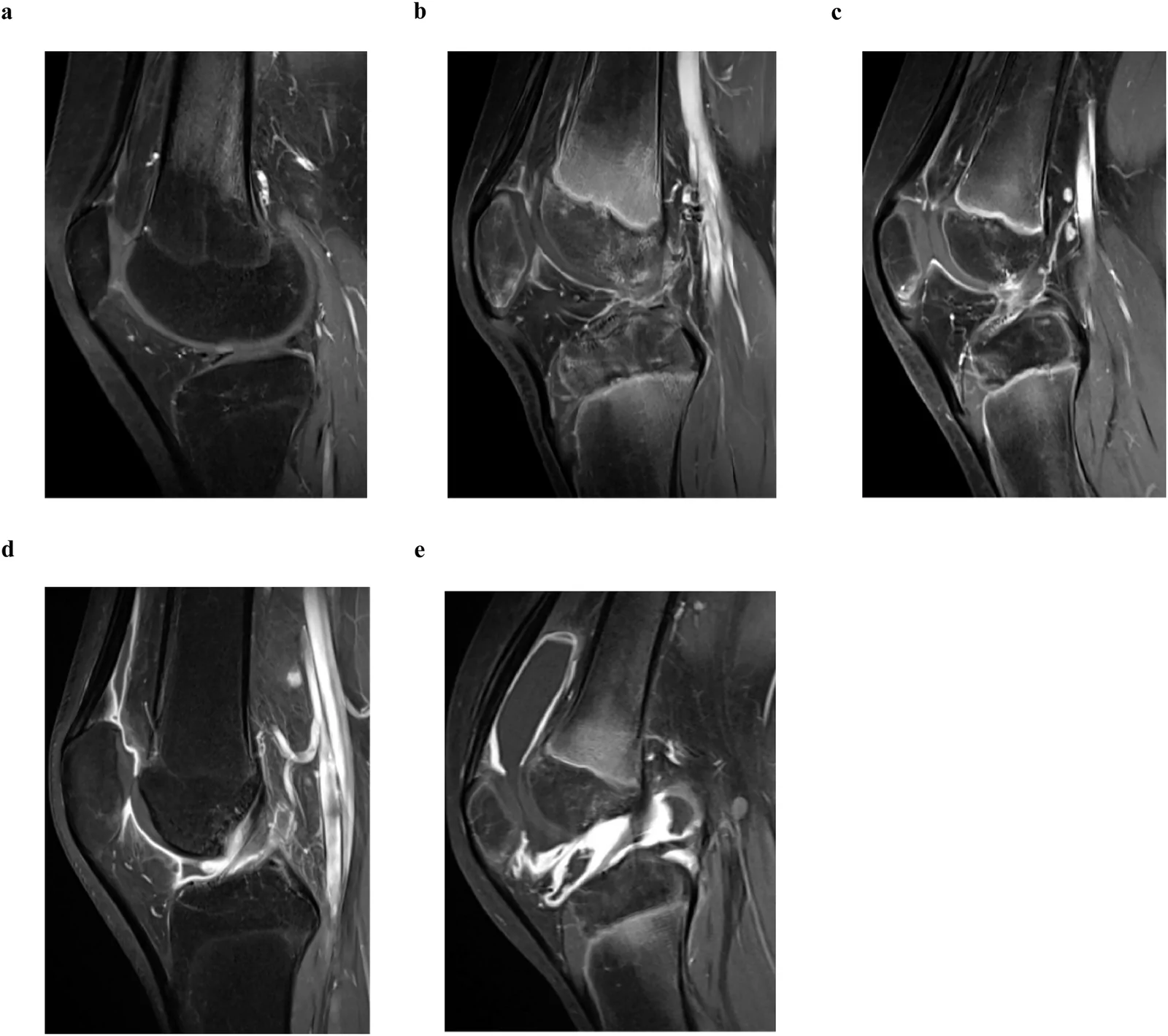
CEMRI atlas with sagittal postcontrast examples of the knee: **a** Grade 0 (none/subtle synovial enhancement); **b**, **c** Grade 1 (mild synovial enhancement); **d** Grade 2 (moderate synovial enhancement); **e** Grade 3 (severe synovial enhancement)

**Fig. 4:**
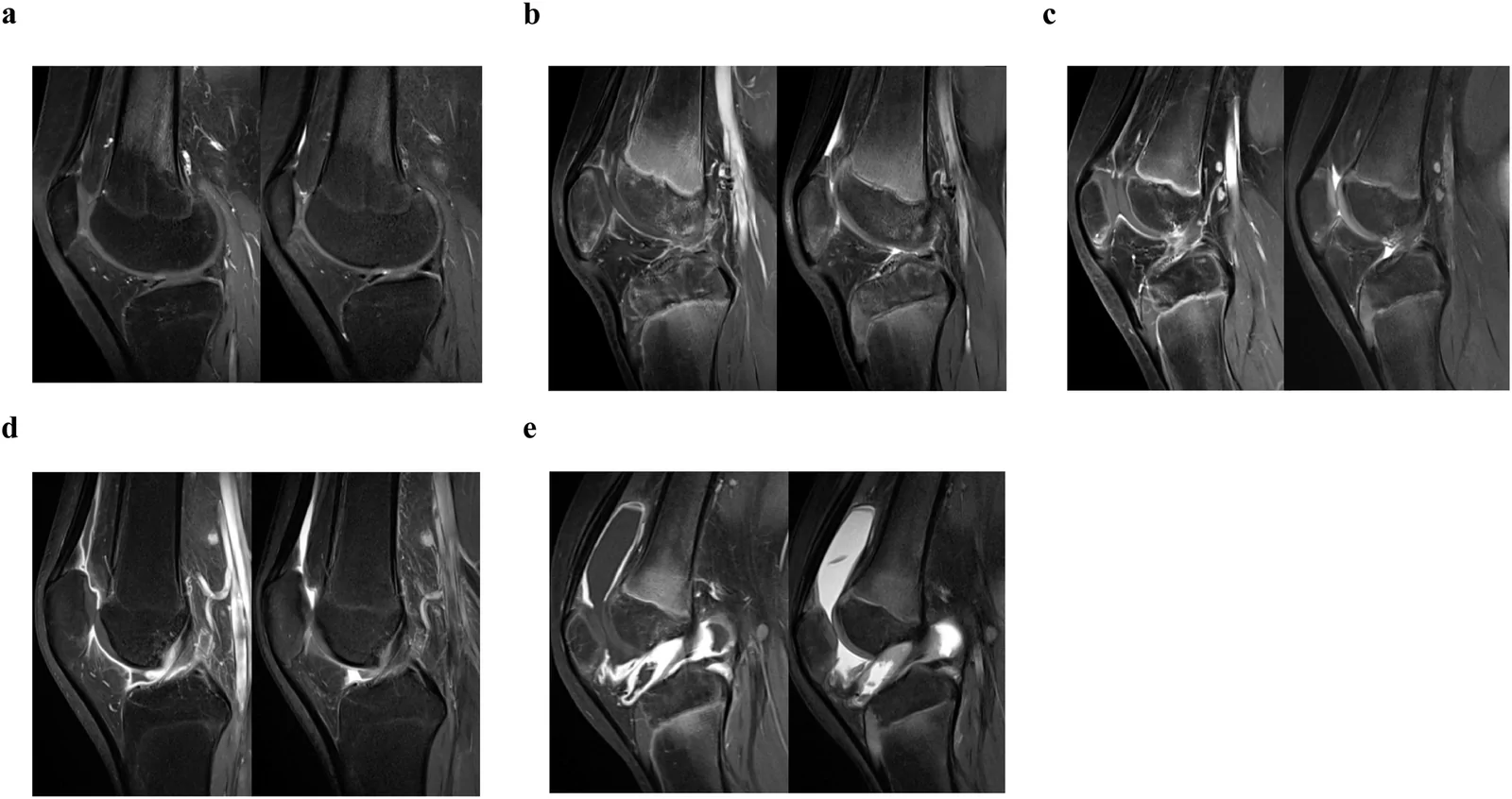
CEMRI + PDFS atlas with paired examples, sagittal images, postcontrast left side image and PDFS right side image: **a** Grade 0 (none/subtle synovial enhancement or effusion); **b**, **c** Grade 1 (mild synovial enhancement/thickening ± effusion); **d** Grade 2 (moderate synovial enhancement/thickening + effusion); **e** Grade 3 (severe synovial enhancement/thickening + effusion)

Based on the T1-w TSE FS pre- and postcontrast images, all MRI examinations were evaluated in a blinded fashion without knowledge of clinical findings, by two radiologists, independently (one paediatric and one musculoskeletal radiologist with 12 and 14 years of experience, respectively). The overall synovial enhancement and degree of inflammation were scored separately, on a 0–3 scale according to a recent study [4]. Overall synovial enhancement was based on the degree of synovial enhancement/synovial thickening and scored as grade 0 (none/subtle); grade 1 (mild); grade 2 (moderate) or grade 3 (severe) (Fig. 3). Overall degree of inflammation was based on a comprehensive evaluation of degree and extent of synovial enhancement and the presence or not of an effusion (defined as ≥ 2 mm at the maximum), and scored as grade 0 (none/subtle synovial enhancement or effusion); grade 1 (mildly increased synovial enhancement/thickening, ± effusion); grade 2 (moderately increased synovial enhancement/thickening, + effusion) or grade 3 (severely increased synovial enhancement/thickening + effusion). An illustrated atlas, using single, sagittal images from five different JIA patients, was devised to standardise grading (Fig. 4).

The MRIs were also scored using elements of the JAMRIS-system [7]; synovial hypertrophy (scored on postcontrast images at six different locations, as 0 (< 2 mm thick), 1 (≥ 2–4 mm) or 2 (> 4 mm) (minimum score 0, maximum 12)) and bone marrow changes suggestive of oedema (scored as grade 0 (none); grade 1 (< 10% of the whole bone volume); 2 (10–25%) or 3 (≥ 25%) (minimum total score 0, maximum 24)). A repeat grading was performed after 6 weeks by both observers, separately, and blinded to previous results.

##### Dynamic CEMRI—quantitative assessment of synovial inflammation.

**Fig. 5:**
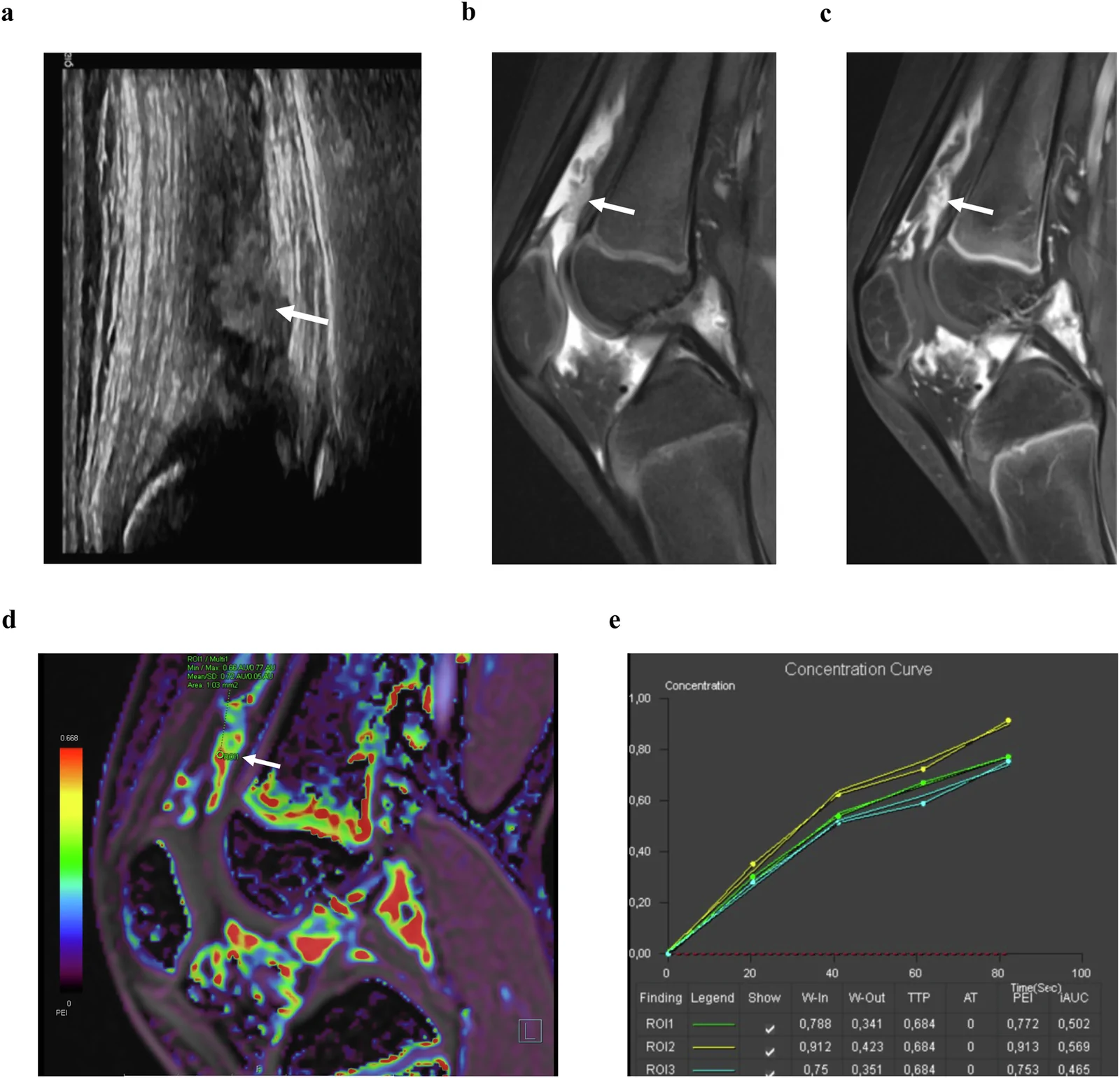
CEMRI in a 7-year-old girl with JIA (same patient as Fig. 1). **a** B-mode, sagittal view showing thickened synovium (arrow). **b** MRI PD FS image and **c** T1 TSE FS postcontrast image, same sagittal plane, showing thickened and enhancing synovium (arrow). **d** Syngo.via® colour peak enhancement intensity (PEI) map with a region of interest (ROI) placed within the enhancing synovium, in the same areas as for CEUS. **e** Three dynamic enhancement curves (0–100 s), representing perfusion in 3 different ROIs; concentration curves display the concentration of the contrast agent in the *y*-axis along time in the *x*-axis

From the sagittal T1-w TSE FS post-gadolinium series, a sagittal section, corresponding to the chosen CEUS view, was identified by the two observers in consensus, and used for the dynamic CEMRI measurements. Using the Syngo.via® Tissue 4D application, the two observers, independently, placed three different circular ROIs (approximately 1mm^2^ area, with the lowest possible standard deviation [SD]) within a vividly enhancing portion of the synovium, guided by the colour PEI map (Fig. 5). PEI, TTP and iAUC were measured.

### Statistical analysis

Continuous data were presented as medians (with interquartile range [IQR]) or means (with standard deviations [SD]) as appropriate, and dichotomous data as absolute/relative frequencies. For categorical variables, intra- and interobserver agreement was assessed using linear weighted kappa (κ) coefficients (95% confidence interval [CI]) and proportion of absolute agreement. A κ of < 0 was considered poor, 0–0.20 slight, 0.21–0.40 fair, 0.41–0.60 moderate, 0.61–0.80 substantial and 0.81–1.00 almost perfect [14]. For comparison purposes, we also employed the intraclass correlation coefficient (ICC), two-mixed, type consistency [15, 16], with 95% CIs (0–0.39 poor, 0.40–0.59 fair, 0.60–0.74 good; and 0.75–1.00 excellent), reflecting relative agreement [17].

Differences in continuous variables were analysed using Bland–Altman plots and 95% limits of agreement (LoAs), reflecting absolute agreement [18]. The quantitative CEUS and CEMRI data were not normally distributed, remaining skewed after log-transformation; thus, non-parametric LoAs were used (2.5th percentile and the 97.5th percentile of the log-differences), reporting the back-transformed values to acquire exponential/ratios for interpretation of bias and LoA. Differences in continuous variables were also analysed using ICCs (two-way mixed; consistency type) as appropriate.

The JAMRIS Score was considered a continuous variable, adding up the scores of the different locations to a maximum of 12 for synovial hypertrophy and 24 for bone marrow oedema (BMO). ICC (two-way mixed; consistency type) was used to test for intra- and interobserver agreement. The statistical analyses were performed using SPSS software (v.30, IBM) or Jamovi® v.2.6.26. A *p* < 0.05 was considered statistically significant.

## Results

Fifty-two patients (35 females) were included, median age 12.4 years (IQR 8.9–15.6). Demographic data and disease characteristics are given in Table 1. The distribution of subjective CEUS and CEMRI-scores for synovial inflammation on a 0–3 scale was balanced, with approximately 8–17 cases in each of the groups.

**Table 1:** Demographic data on the study population

Patients	
*n* (%)	52 (100)
Sex, female, *n* (%)	35 (67.4)
Age, years, Mdn (IQR)	12.4 (8.9–15.6)
Height, cm, Mdn (IQR)	149.0 (129.0 –161.0)
Weight, kg, Mdn (IQR)	39.7 (26.1–56.6)
BMI, Mdn (IQR)	18.3 (16.1–21.8)
JIA subtypes, *n* (%)	52 (100)
Systemic	2 (3.8)
Oligoarthritis persistent	30 (57.7)
Oligoarthritis extended	5 (9.6)
Polyarthritis FR negative	6 (11.5)
Polyarthritis FR positive	1 (1.9)
Psoriatic arthritis	4 (7.7)
Enthesitis-related arthritis	4 (7.7)
Age at JIA diagnosis, years, Mdn (IQR)	5.0 (2.0–8.0)
Duration of JIA’s disease, years, Mdn (IQR)	6.0 (3.3–9.0)
Right knee studied, *n* (%)	28 (53.8)

*Mdn* median, *IQR* interquartile range, *BMI* body mass index

### Contrast-enhanced ultrasound (CEUS)

#### Subjective grading of synovial inflammation; intra- and interobserver agreement

Based on the videoclips, assessment of the degree of synovial contrast enhancement on a 0–3 scale performed almost perfectly for both observers, with kappa values of 0.89 (95% CI 0.81–0.98) and 0.90 (95% CI 0.83–0.98), for the two readers, respectively (Table 2). Agreement between observers was almost perfect with a kappa value of 0.85 (95% CI 0.76–0.93) and an absolute agreement of 0.83. Using ICC, the interobserver agreement was excellent (0.96; 95% CI 0.93–0.98).

**Table 2:** Subjective CEUS-scoring assessment of synovial inflammation of the knee joint in 52 children with JIA, based on reading of the videoclips taken by observer one

CEUS-feature	Intraobserver		Interobserver		
	Linear kappa value (95% CI)	ICC (95% CI)	Linear kappa value (95% CI)	Proportion absolute agreement (95% CI)	ICC (95% CI)
Degree of synovial contrast enhancement on a 0–3 scale	0.89 (0.81–0.98)—R1 0.90 (0.84–0.98)—R2	0.97 (0.95–0.98)—R1 0.97 (0.96–0.99)—R2	0. 85 (0.76–0.94)	0.83 (0.69–0.91)	0.96 (0.93–0.98)

R1 = reader one, R2 = reader two

Linear weighted kappa values and ICCs for intra- and interobserver agreement and proportion of absolute agreement for interobserver variation are reported

#### Dynamic CEUS: quantitative measurement of synovial inflammation; interobserver agreement

Measurements of PE and WiAUC performed well, with ICCs of 0.99 (95% CI 0.99–1.00) and 0.97 (95% CI 0.95–0.98), respectively, while TTP had a moderate interobserver agreement, with an ICC of 0.66 (95% CI 0.40–0.80).

**Fig. 6:**
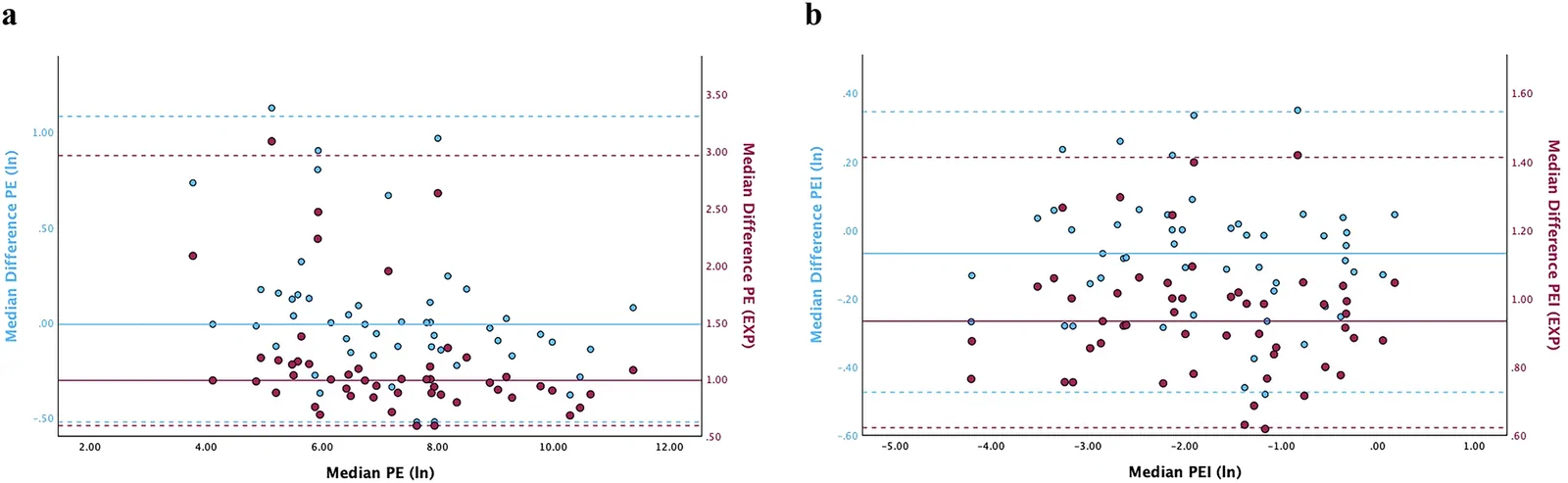
Non-parametric Bland–Altman plot of interobserver agreement: **a** for peak enhancement (PE) on CEUS, the primary *y*-axis (left) displays the difference between observers on a natural log (ln) scale, whilst the secondary *y*-axis (right) displays the corresponding back-transformed ratios (EXP). The solid horizontal line (blue) indicates the median bias of −0.01 (corresponding back-transformed bias: 0.99, solid red line), the blue stippled lines indicate the 95% LoAs from −0.53 to 1.08 (corresponding back-transformed ratio LoAs: 0.59–2.96, stippled red lines); **b** for peak enhancement intensity (PEI) on CEMRI, the primary *y*-axis (left) displays the difference between observers on a natural log (ln) scale, whilst the secondary *y*-axis (right) displays the corresponding back-transformed ratios (EXP). The solid horizontal line (blue) indicates the median bias of −0.07 (corresponding back-transformed bias: 0.93, solid red line), the blue stippled lines indicate the 95% LoAs from −0.47 to 0.34 (corresponding back-transformed ratio LoAs: 0.62–1.41, stippled red lines)

For PE, the interobserver median ratio using a non-parametric Bland–Altman analysis with back-transformation, was 0.99, indicating negligible systematic bias between observers, however, the LoAs (0.59–2.96) were wide, demonstrating poor interobserver reproducibility (Fig. 6). Corresponding figures for WiAUC were 0.94 with 95% LoA of 0.39–3.71, and for TTP, 0.96 with 95% LoA of 0.40–1.81.

### MRI

#### Subjective grading of synovial inflammation; intra- and interobserver agreement

Assessment of overall synovial inflammation on a 0–3 scale showed almost perfect agreement for both observers, with kappa values of 0.91 (95% CI 0.83–0.99) and 0.96 (95% CI 0.91–1.00), respectively (Table 3). The interobserver agreement was 0.89 (95% CI 0.81–0.98), with an absolute agreement of 0.88. Based on ICC, the interobserver agreement was excellent (0.97; 95% CI 0.95–0.98).

**Table 3:** Subjective MRI-scoring assessment of the synovial inflammation of the knee joint of 52 children with JIA, based on T1-w TSE FS pre and postcontrast images

MRI-features	Intraobserver		Interobserver		
	Linear kappa value (95% CI)	ICC (95% CI)	Linear kappa value (95% CI)	Proportion absolute agreement (95% CI)	ICC (95% CI)
Overall synovial inflammation on a 0–3 scale	0.91 (0.83–0.99)—R1 0.96 (0.91–1.00)—R2	0.97 (0.96–0.99)—R1 0.99 (0.98 –0.99)—R2	0.89 (0.81–0.98)	0.88 (0.76–0.95)	0.97 (0.95–0.98)
Overall degree of inflammation, including effusion on a 0–3 scale	0.94 (0.88–1.00)—R1 0.90 (0.81–0.98)—R2	0.98 (0.97–0.99)—R1 0.97 (0.94–0.98)—R2	0.92 (0.85–1.00)	0.92 (0.81–1.00)	0.98 (0.96–0.99)

R1 = reader one, R2 = reader two

Linear weighted kappa values and ICCs for intra- and interobserver agreement and proportion of absolute agreement for interobserver variation are reported

Grading overall inflammation of the knee joint based on synovial enhancement, thickening and effusion also performed very well, with kappa values of 0.94 (95% CI 0.88–1.00) and 0.90 (95% CI 0.81–0.98) for each observer, respectively. The interobserver agreement was 0.92 (95% CI 0.85–1.00), with 0.92 of absolute agreement (Table 3). The ICC for interobserver agreement was almost perfect (0.98; 95% CI 0.96–0.99).

#### Subjective grading using components from the JAMRIS score, inflammatory domain

Assessing synovial hypertrophy for each of the six locations, according to the JAMRIS system, performed well both between and for the same observers (Table 4). For the total score synovial hypertrophy on a 0–12 scale, the agreement was almost perfect for intra-observers, with ICCs of 0.97 (95% CI 0.96–0.99) and 0.99 (95% CI 0.99–1.00). The interobserver agreement was 0.97 (95% CI 0.95–0.98).

**Table 4:** Juvenile arthritis MRI scoring (JAMRIS) system for the knee—inflammatory domain with synovial hypertrophy and bone marrow oedema: median with interquartile range (IQR) and range measures for both readers

JAMRIS	Reader 1 Median (IQR) *Range (Min–Max)*	Reader 2 Median (IQR) *Range (Min–Max)*	*p*-value^a^	ICC intraobserver	ICC interobserver
Total score synovial hypertrophy (0–12)	3.50 (0.00–8.00) *(0–12)*	3.00 (0.00–8.00) *(0–12)*	0.940	0.97 (0.96–0.99)—R1 0.99 (0.99–1.00)—R2	0.97 (0.95–0.98)
Total score bone marrow oedema (0–24)	0.00 (0.00–1.00) *(0–7)*	0.00 (0.00–0.00) *(0–3)*	**0.041**	0.88 (0.79–0.93)—R1 0.92 (0.86–0.95)—R2	0.65 (0.39–0.80)

R1 = reader one, R2 = reader two

Intraclass correlation coefficient (ICC) for intra- and inter-observers was also assessed in the study population (52 patients with JIA; 35 girls)

^a^ Mann–Whitney test

Bold = *p*-value < 0.05 (not italic); Italic = range values (min-max)

BMO was identified in 9/52 and 19/52 patients, respectively, by the two readers, yielding almost perfect intraobserver variation of 0.88 (95% CI 0.79–0.99) and 0.92 (95% CI 0.86–0.95) for the two readers, respectively. The interobserver agreement was moderate with ICC of 0.65 (95% CI 0.39–0.80) (Table 4).

#### Dynamic CEMRI: semi-quantitative measurement of synovial inflammation; interobserver agreement

Measurements of PEI and iAUC had excellent interobserver agreement with ICCs of 0.99 (95% CI 0.98–0.99) and 0.98 (95% CI 0.97–0.99), respectively, while TTP had a good agreement with an ICC of 0.76 (95% CI 0.59–0.86).

For PEI, the interobserver median ratio was 0.93, indicating a small systematic bias between observers of 7%, however, the 95% LoAs were wide (0.62–1.41) (Fig. 6). For the iAUC, differences between readers were normally distributed, thus, standard Bland–Altman analysis was performed, showing a mean difference between readers of −0.01, with fair 95% LoA of −0.10 to 0.07.

The TTP was considered a categorical variable, as only a few fixed values were allowed by the application, yielding a moderate interobserver agreement, kappa values of 0.58 (95% CI 0.39–0.77) and an absolute agreement of 0.69.

## Discussion

We have shown that subjective grading of synovial inflammation on a 0–3 scale performed well, both within and between observers, for CEUS and CEMRI. Interobserver analysis for quantitative measurements revealed overall high ICCs, suggesting a very good ability to distinguish between subjects. However, the LoAs, most of which were wide, indicate that the interobserver precision may be insufficient for tracking subtle changes in a single subject over time.

Evaluating the degree of synovial inflammation with ultrasound and/or CEMRI is important for disease management in children with JIA [19–21]. A recent review provides validated scoring systems for both modalities, as well as published reference values for the amount of joint fluid and synovial thickness [6]. The potential of dynamic CEUS and CEMRI for grading arthritis has been evaluated; however, limited studies have assessed their reproducibility and precision in children [22–25]. In particular, no study evaluating both CEUS and CEMRI based on the same cohort and performed on the same day exists. In a study from 2010, including 22 patients with involvement of the wrist or hip, the reproducibility of dynamic MRI-measurements was reportedly excellent when based on ICCs; however, without elaborating on the results using additional statistical tests [26]. Bennett et al [27,28] in another study of 11 patients treated with intra-articular glucocorticoid injections, found that quantitative MR-measurements were reliable in the assessment of synovial inflammation, using a Bland–Altman approach.

Knowledge of a method’s reliability is crucial for the correct interpretation of changes in a specific imaging marker over time. We used two complementary statistical methods: an ICC, “two-mixed type” measuring the overall consistency (reliability) of the markers, and 95% LoA indicating the range where 95% of paired differences are expected to fall. LoA, which shows the clinical relevance of measurement differences, shares similarities with the clinical measure “minimal detectable change” (MDC), also known as “smallest detectable change” (SDC), using a 95% confidence interval, which is similar in calculation to LoA but focused on true change rather than method agreement [15]. For example, a LoA of 0.62–1.41 as found for PEI (CEMRI) indicates that measurements of the same subject could vary from −38% to 41% between observers. While the high ICC of 0.99 suggests excellent discriminative ability across the scale used, the wide LoAs indicate that the precision is poor for clinical monitoring. The conflicting statistical results can in part be explained by the large arbitrary scales used for both CEMRI (signal intensity, SI) and CEUS (Linear Units, a.u.), reflecting the inherent limitations of manual ROI placement on high-intensity linear scales.

The subset of JAMRIS markers examined performed well for synovial hypertrophy total score, supporting previous results [7]. More recently, Handa et al [29], in a study of 35 patients with JIA, reported moderate to high intraobserver agreement for synovial thickness measurements, with concordance correlation coefficients (CCC) from 0.50 to 0.89 for six locations used in the JAMRIS scoring system. Interobserver agreement was low, with a CCC of 0.47, averaged over six locations, particularly for unenhanced images. In contrast, Verkuil et al [30] demonstrated excellent inter- and intraobserver agreement (ICC) for both unenhanced (double inversion recovery (DIR)) and CEMR images for the JAMRIS-compartments (26 children with JIA, of whom 13 had active inflammation). However, a BA-plot showed wide LoAs, from 1.4 to 1.6 mm for the four readers, reflecting low precision.

In our study, assessment of BMO performed well within observers; however, the interobserver agreement was moderate, with significant differences between the two observers. In comparison, Hemke demonstrated a high ICC of 0.86 and no significant differences between readers [7]. These discrepancies reflect challenges of defining and diagnosing BMO. Recent studies have shown that around 50% of healthy children/adolescents have at least one major BMO-like change in the appendicular skeleton, particularly around knee joints, which, in a clinical setting, can be mistaken for pathology [31–34]. According to JAMRIS [7], changes suggestive of BMO are defined as lesions within the trabecular bone, with ill-defined margins and high signal intensity on T2-weighted fat-saturated images and low signal intensity on T1-weighted images, without mention of what is considered normal variation. Although using their definition, our perception of high T2-signal beyond normality might have differed. Moreover, our cohort included a higher number of young children as compared to the JAMRIS cohort [35, 36].

We acknowledge some limitations to our study. First, the cohort was relatively small; however, the distribution of scores along the 0–3 scale was balanced, reducing the risk of bias. Second, differences in the placement of the ROIs might have influenced the results. Third, the atlases devised for subjective scoring included a few images from the studied cohort, images not used for later scoring. Lastly, intraobserver agreement for quantitative MRI data was not performed.

## Conclusion

Subjective grading of synovial inflammation on a 0–3 scale in JIA patients with knee involvement performed well, both within and between observers, for CEUS and CEMRI. Both methods have excellent relative reliability (ICC) for quantitative measurements, meaning that they work at a group level; however, their absolute interobserver precision (LoA) is poor.

## Supplementary information

Supplementary information

## Abbreviations

BMO: Bone marrow oedema
CEMRI: Contrast-enhanced MRI
CEUS: Contrast-enhanced ultrasound
iAUC: Initial area under the curve
ICC: Intraclass correlation coefficient
JAMRIS: Juvenile arthritis MRI scoring
JIA: Juvenile idiopathic arthritis
LoA: Limits of agreement
PE: Peak enhancement
PEI: Peak enhancement intensity
ROI: Region of interest
TTP: Time to peak
WiAUC: Wash-in area under the curve

## References

[1] Gabriel SE, Michaud K (2009) Epidemiological studies in incidence, prevalence, mortality, and comorbidity of the rheumatic diseases. Arthritis Res Ther 11:229 19519924 10.1186/ar2669 PMC2714099

[2] Thierry S, Fautrel B, Lemelle I, Guillemin F (2014) Prevalence and incidence of juvenile idiopathic arthritis: a systematic review. Joint Bone Spine 81:112–117 24210707 10.1016/j.jbspin.2013.09.003

[3] Prakken B, Albani S, Martini A (2011) Juvenile idiopathic arthritis. Lancet 377:2138–2149 21684384 10.1016/S0140-6736(11)60244-4

[4] Arvidsson LZ, Smith HJ, Flato B, Larheim TA (2010) Temporomandibular joint findings in adults with long-standing juvenile idiopathic arthritis: CT and MR imaging assessment. Radiology 256:191–200 20574096 10.1148/radiol.10091810

[5] Glerup M, Rypdal V, Arnstad ED et al (2020) Long-term outcomes in juvenile idiopathic arthritis: eighteen years of follow-up in the population-based Nordic juvenile idiopathic arthritis cohort. Arthritis Care Res 72:507–516 10.1002/acr.23853 30762291

[6] Costa Dias S, Habre C, Di Paolo PL et al (2026) ESR Essentials: juvenile idiopathic arthritis; what every radiologist needs to know—practice recommendations by the European Society of Paediatric Radiology. Eur Radiol 36:1261–1271 10.1007/s00330-025-11891-9 PMC12953336 40828237

[7] Hemke R, van Rossum MA, van Veenendaal M et al (2013) Reliability and responsiveness of the juvenile arthritis MRI scoring (JAMRIS) system for the knee. Eur Radiol 23:1075–1083 23085866 10.1007/s00330-012-2684-y

[8] Hemke R, Tzaribachev N, Nusman CM, van Rossum MAJ, Maas M, Doria AS (2017) Magnetic resonance imaging (MRI) of the knee as an outcome measure in juvenile idiopathic arthritis: an OMERACT reliability study on MRI scales. J Rheumatol 44:1224–1230 28572469 10.3899/jrheum.160821

[9] Petty RE, Southwood TR, Manners P et al (2004) International League of Associations for Rheumatology classification of juvenile idiopathic arthritis: second revision, Edmonton, 2001. J Rheumatol 31:390–392 14760812

[10] European Medicines Agency (2026) Available via https://www.ema.europa.eu/en/homepage. Accessed 28 Jan 2026

[11] Dias SC, Carvalho DC, Castro M et al (2025) Knee-ultrasound: reference values for the amount of joint fluid and synovial appearances in healthy children and adolescents. Pediatr Radiol 55:1127–1137 40314755 10.1007/s00247-025-06243-0 PMC12119740

[12] Gong Y, Huang Y, Su Y, He J, Chen S (2021) Value of contrast-enhanced ultrasonography in evaluating rheumatoid arthritis: preliminary research based on an animal model. Med Sci Monit 27:e931327 34172694 10.12659/MSM.931327 PMC8243804

[13] Klauser A, Demharter J, De Marchi A et al (2005) Contrast enhanced gray-scale sonography in assessment of joint vascularity in rheumatoid arthritis: results from the IACUS study group. Eur Radiol 15:2404–2410 16132921 10.1007/s00330-005-2884-9

[14] Landis JR, Koch GG (1977) The measurement of observer agreement for categorical data. Biometrics 33:159–174 843571

[15] de Vet HC, Terwee CB, Knol DL, Bouter LM (2006) When to use agreement versus reliability measures. J Clin Epidemiol 59:1033–1039 16980142 10.1016/j.jclinepi.2005.10.015

[16] Kottner J, Audige L, Brorson S et al (2011) Guidelines for reporting reliability and agreement studies (GRRAS) were proposed. Int J Nurs Stud 48:661–671 21514934 10.1016/j.ijnurstu.2011.01.016

[17] Hallgren KA (2012) Computing inter-rater reliability for observational data: an overview and tutorial. Tutor Quant Methods Psychol 8:23–34 22833776 10.20982/tqmp.08.1.p023 PMC3402032

[18] Bland JM, Altman DG (1999) Measuring agreement in method comparison studies. Stat Methods Med Res 8:135–160 10501650 10.1177/096228029900800204

[19] Malattia C, Tolend M, Mazzoni M et al (2020) Current status of MR imaging of juvenile idiopathic arthritis. Best Pract Res Clin Rheumatol 34:101629 33281052 10.1016/j.berh.2020.101629

[20] Mazzoni M, Pistorio A, Magnaguagno F et al (2023) Predictive value of magnetic resonance imaging in patients with juvenile idiopathic arthritis in clinical remission. Arthritis Care Res 75:198–205 10.1002/acr.24757 PMC10087925 34286915

[21] De Lucia O, Ravagnani V, Pregnolato F et al (2018) Baseline ultrasound examination as possible predictor of relapse in patients affected by juvenile idiopathic arthritis (JIA). Ann Rheum Dis 77:1426–1431 29437586 10.1136/annrheumdis-2017-211696

[22] Ntoulia A, Barnewolt CE, Doria AS et al (2021) Contrast-enhanced ultrasound for musculoskeletal indications in children. Pediatr Radiol 51:2303–2323 33783575 10.1007/s00247-021-04964-6

[23] Nusman CM, Hemke R, Lavini C et al (2017) Dynamic contrast-enhanced magnetic resonance imaging can play a role in predicting flare in juvenile idiopathic arthritis. Eur J Radiol 88:77–81 28189212 10.1016/j.ejrad.2017.01.003

[24] Doria AS, Kiss MH, Lotito AP et al (2001) Juvenile rheumatoid arthritis of the knee: evaluation with contrast-enhanced color Doppler ultrasound. Pediatr Radiol 31:524–531 11486809 10.1007/s002470100474

[25] Workie DW, Graham TB, Laor T et al (2007) Quantitative MR characterization of disease activity in the knee in children with juvenile idiopathic arthritis: a longitudinal pilot study. Pediatr Radiol 37:535–543 17401557 10.1007/s00247-007-0449-6

[26] Malattia C, Damasio MB, Basso C et al (2010) Dynamic contrast-enhanced magnetic resonance imaging in the assessment of disease activity in patients with juvenile idiopathic arthritis. Rheumatology 49:178–185 19995859 10.1093/rheumatology/kep343

[27] Bennett JL, Wood A, Smith N et al (2019) Can quantitative MRI be used in the clinical setting to quantify the impact of intra-articular glucocorticoid injection on synovial disease activity in juvenile idiopathic arthritis? Pediatr Rheumatol Online J 17:74 31752877 10.1186/s12969-019-0377-7 PMC6873560

[28] Bunce C (2009) Correlation, agreement, and Bland–Altman analysis: statistical analysis of method comparison studies. Am J Ophthalmol 148:4–6 19540984 10.1016/j.ajo.2008.09.032

[29] Handa A, Bedoya MA, Iwasaka-Neder J, Johnston PR, Lo MS, Bixby SD (2024) Measuring synovial thickness on knee MRI in pediatric patients with arthritis: is contrast necessary? Pediatr Radiol 54:988–1000 38641735 10.1007/s00247-024-05929-1

[30] Verkuil F, Hemke R, van Gulik EC et al (2022) Double inversion recovery MRI versus contrast-enhanced MRI for evaluation of knee synovitis in juvenile idiopathic arthritis. Insights Imaging 13:167 36264355 10.1186/s13244-022-01299-0 PMC9584003

[31] Zadig P, von Brandis E, d’Angelo P et al (2022) Whole-body MRI in children aged 6–18 years. Reliability of identifying and grading high signal intensity changes within bone marrow. Pediatr Radiol 52:1272–1282 35445816 10.1007/s00247-022-05312-y PMC9192437

[32] von Brandis E, Jenssen HB, Avenarius DFM et al (2022) Automated segmentation of magnetic resonance bone marrow signal: a feasibility study. Pediatr Radiol 52:1104–1114 35107593 10.1007/s00247-021-05270-x PMC9107442

[33] Zadig PK, von Brandis E, Flato B et al (2022) Whole body magnetic resonance imaging in healthy children and adolescents: bone marrow appearances of the appendicular skeleton. Eur J Radiol 153:110365 35617871 10.1016/j.ejrad.2022.110365

[34] von Brandis E, Zadig PK, Avenarius DFM et al (2022) Whole body magnetic resonance imaging in healthy children and adolescents. Bone marrow appearances of the axial skeleton. Eur J Radiol 154:110425 35843014 10.1016/j.ejrad.2022.110425

[35] Person A, Janitz E, Thapa M (2021) Pediatric bone marrow: normal and abnormal MRI appearance. Semin Roentgenol 56:325–337 34281683 10.1053/j.ro.2021.05.002

[36] Tanturri de Horatio L, Zadig PK, von Brandis E, Ording Muller LS, Rosendahl K, Avenarius DFM (2023) Whole-body MRI in children and adolescents: can T2-weighted Dixon fat-only images replace standard T1-weighted images in the assessment of bone marrow? Eur J Radiol 166:110968 37478654 10.1016/j.ejrad.2023.110968

