# Supplementary material for: Reliability of CEUS and MRI for grading knee-joint inflammation in juvenile idiopathic arthritis
Source: Eur Radiol. 2026 May 15;36(10):7379–90. doi: 10.1007/s00330-026-12627-z (PMC13574867; doi:10.1007/s00330-026-12627-z)
Supplement: Supplementary file 1 [file 330_2026_12627_MOESM1_ESM.pdf]

Reliability of CEUS and MRI for grading knee-joint inflammation in Juvenile Idiopathic Arthritis

ELECTRONIC SUPPLEMENTARY MATERIAL

Eur Radiol (2026) Costa Dias S, Ferreira CC, Santos I, et al.

Electronic Supplemental Material – MRI Protocol, “Siemens Magnetom Vida 3T” with Knee coil. 30º Flexion.

Sequence	Plane	TR (ms)	TE (ms)	TI (ms)	Averages	FOV read (mm)	FOV phase	Number slices	Slice Thickness (mm)	Flip angle	Gap (mm)	Image Matrix	Band width (Hz/Px)	Acquisition time	Turbo factor	Acceleration mode	Acceleration factor	Others
**PD TSE FS**	Sagittal	3010	33	---	1	160	100%	32	3	180	0.3	269*384	181	1min31 s	9	SMS	2	SPAIR<br>PE: H>>F<br>Oversampling: 70%
	Axial	4160	38	---	1	160	100%	40	3	180	0.3	269*384	148	1min44 s	9	SMS	2	SPAIR<br>PE: R>>L<br>Oversampling: 40%
	Coronal	3010	33	---	1	160	100%	38	3	150	0.3	269*384	181	1min30 s	7	SMS	2	SPAIR<br>PE: R>>L<br>Oversampling: 30%
**T1 TSE**	Coronal	718	9.9	---	1	160	100%	38	3	150	0.3	314*448	228	1min09 s	3	SMS	2	PE: R>>L<br>Oversampling: 50%
**T1 TSE FS pre - Gd**	Sagittal	500	9	---	2	160	100%	32	3	150	0.3	280*400	227	2min51 s	3	SMS	2	PE: H>>F<br>Fat suppression<br>Oversampling: 70%
**T1 vibe Dixon fatsat pre contrast**	Sagittal	3.91	1.23 and 2.46	---	1	160	100%	80	1.3	9	0	128*128	1180	23s each measurement	---	CAIPIRINHA	2	5 measurements<br>Slice oversampling: 100%<br>PE: P>>A<br>DIXON method
**T1 vibe Dixon fatsat caipirinha first 2 min (isotropic)**	Sagittal	3.91	1.23 and 2.46	---	1	160	100%	80	1.3	9	0	128*128	1180	23s each measurement	---	CAIPIRINHA	2	5 measurements<br>Slice oversampling: 100%<br>PE: P>>A<br>DIXON method
**T1 TSE FS post - Gd**	Sagittal	500	9	---	2	160	100%	32	3	150	0.3	280*400	227	2min51 s	3	SMS	2	PE: H>>F<br>Fat suppression<br>Oversampling: 70%
**T1 TSE FS post - Gd**	Axial	752	8.9	---	2	160	100%	40	3	150	0.3	280*400	228	2min35 s	3	SMS	2	PE: R>>L<br>Fat suppression<br>Oversampling: 5%
